# New Record of *Brachylaima* sp. (Digenea: Brachylaimidae) from a Stray Dog in North Iran

**Published:** 2017

**Authors:** Iraj MOBEDI, Mahdi FAKHAR, Malik IRSHADULLAH, Bahman RAHIMI-ESBOEI, Shirzad GHOLAMI, Natalia FRAIJA-FERNÁNDEZ

**Affiliations:** 1. Dept. of Parasitology and Mycology, School of Public Health, Tehran University of Medical Sciences, Tehran, Iran; 2. Toxoplasmosis Research Center, Department of Parasitology and Mycology, School of Medicine, Mazandaran University of Medical Sciences, Sari, Iran; 3. Dept. of Zoology, Aligarh Muslim University, Aligarh, India; 4. Marine Zoology Unit, Cavanilles Institute of Biodiversity and Evolutionary Biology, Science Park, University of Valencia, Valencia, Spain

**Keywords:** Stray dog, Zoonosis, Digenea, *Brachylaima*, Iran

## Abstract

**Background:**

Stray dogs are considered potential reservoirs for zoonotic diseases. Previous helminthic surveys in Iran, have accounted for mainly species of nematodes and cestodes, and rarely digeneans.

**Methods:**

We accessed 42 car-crashed stray dogs from the Farah Abad Region in the Mazandaran Province (North Iran) between Oct 2012 and Dec 2013, to be inspected for parasites. Helminths were collected from the intestine and they were morphologically studied.

**Results:**

We found five adult digeneans from the family Brachylaimidae, identified as *Brachylaima* sp. Worms were assigned to the genus based on the shape of the body, the position of genital pore, cirrus sac and testes, and the extension of the vitellarium. Absence of additional information on the developmental stages of the parasite precluded its specific identification. As the geographic distribution of species of *Brachylaima* is restricted to the Mediterranean region, we raise the hypothesis that dogs may become infected with parasites through the consumption of helicid snails when searching for food on the street.

**Conclusion:**

This is the second report of a species of *Brachylaima* in Iran and the third digenean species from stray dogs in the area. We want to raise the attention of researchers to helminthic surveys in potential zoonotic reservoirs like stray dogs.

## Introduction

Stray dogs, *Canis familiaris*, in Iran are considered potential reservoirs for zoonotic diseases and a risk to public health (1, 2). The large population of stray dogs in Iran generates hygienic issues affecting the public perception of dogs and making policies for prevention and control of diseases difficult to accomplish (1). Over the last decade, a significant burden, i.e., >80%, of zoonotic species, mainly nematodes, and cestodes, have been reported in stray dogs in Iran, believed to become infected by consuming water, soil or food with helminth larvae (1-4). Interestingly, only one species of digeneans of the family Diplostomidae (*Alaria alata*), one of the family Heterophyidae (*Ascocotyle sinoecum*), and one of the Brachylaimidae (*Brachylaima* sp.) have been reported in these surveys (1, 5), highlighting the low prevalence of this group of helminths. Nevertheless, in other geographic regions, dogs have been found to harbour several species of digeneans, from which the families Echinostomatidae*,* Heterophyidae*,* Opisthorchiidae, and Paragonimidae have a major species representation (6). Therefore, the routinely parasitic surveys in zoonotic species, like stray dogs, should be considered as of primary importance.

**Fig. 1:**
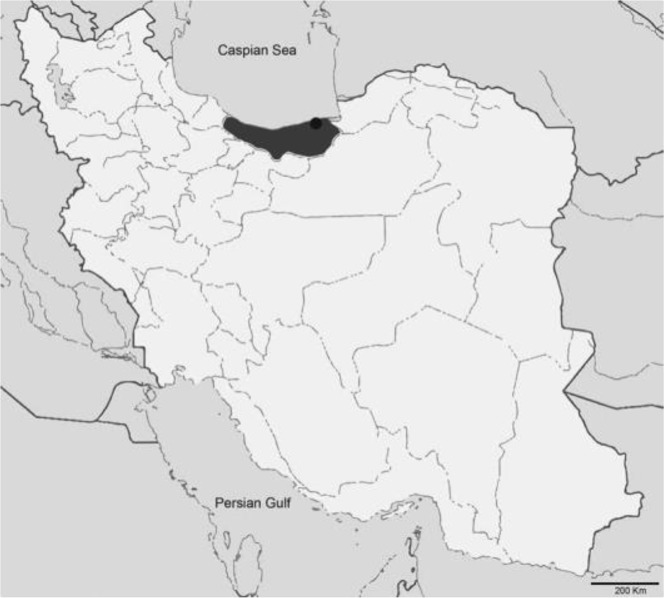
Map of Iran and collection site of the specimens of *Brachylaima* sp. from a stray dog. The Mazandaran province is colored in dark grey and the Farah Abad region is indicated by a black circle.

The Mazandaran Province is located in the north of Iran and the southern coast of the Caspian Sea (Fig. 1). This is one of the most densely populated provinces in Iran and has a changing climate from mild to humid, with variable rates of rainfall throughout the year (7). In this study, we provide evidence on the occurrence of a digenean from the Brachylaimidae isolated from a stray dog in the Farah Abad region in the central part of the Mazandaran Province.

## Materials and Methods

Between Oct 2012 and Dec 2013, 42 car-crashed stray dogs, *Canis familiaris,* were collected in the Mazandaran Province, Northern Iran. Animals were transported to the Laboratory at the School of Medicine in the Mazandaran University of Medical Sciences for necropsy.

The intestine of each animal was separated from the rest of the organs and inspected for parasites. Each intestinal content was filtered over a sieve of 180 μm and 250 μm meshes to collect helminths. Worms were isolated, washed with saline, flattened with a light pressure over the cover glass and fixed in lactophenol. Parasites were stained with carmine, dehydrated and cleared in xylene. Each specimen was mounted in slides with Canada balsam.

Morphometric analyses of stained specimens were performed on an Olympus BX41 microscope connected to an Olympus Dp12 Digital camera calibrated with an eyepiece micrometer. Drawings of each individual were done under an optical microscope with Camera Lucida.

## Results

**Fig. 2:**
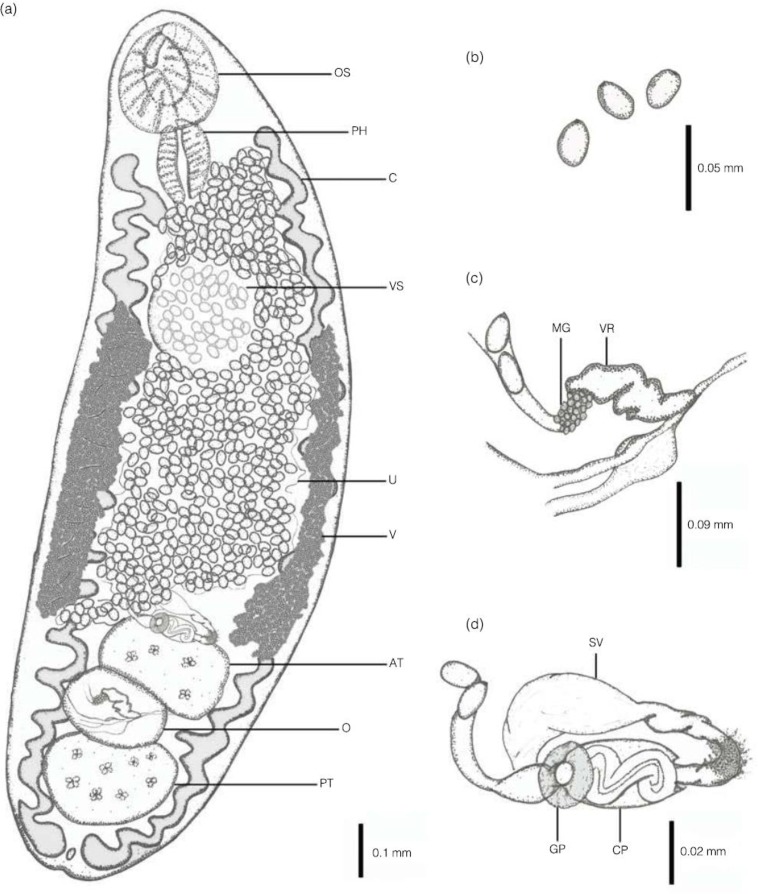
*Brachylaima* sp. from a stray dog, *Canis familiaris*. (a) Ventral view. (b) Detail of eggs. (c) Detail of female reproductive system. (d) Detail of male reproductive system. Scale bars are indicated for each image. Abbreviations: AT, Anterior testis; C, caeca; CP, Cirrus pouch; GP, Genital pore; MG, Mehlis’s gland; O, Ovary; OS, Oral sucker; PH, Pharynx; PT, posterior testis; SV, Seminal vesicle; U, Uterus; V, Vitelline follicles; VR, Vitel-line reservoir; VS, Ventral sucker

Five adult digeneans were collected from the intestine of 1 out of the 42 stray dogs surveyed in the Farah Abad district in the Mazandaran Province (Fig. 2). The specimens found were keyed down to the family Brachylaimidae and the genus *Brachylaima* based on the shape of the body, the position of the genital pore, cirrus sac, and testes, and the extension of the vitellarium (Fig. 2). All the material reported from this study is deposited in the Iranian National Parasitology Museum at the Faculty of Medicine at the University of Tehran, Iran (ID. 829/9.1393), and it is available upon request.

### Morphological description

Observations and measurements based on 5 whole-mounted specimens. Measurements (length × width) are shown as the range, with the mean in parenthesis followed by the standard deviation, and are expressed in micrometers (Table 1, Fig. 2).

**Table 1:** Measurements (in μm) of adult worms (n=5) of *Brachylaima* sp. isolated from a stray dog, *Canis familiaris*, in the Farah Abad region, Mazandaran Province (Iran)

***Variable***		***Mean±SD***	***Range***
Worm body			
	Length	2,214 ± 991.4	1,490 – 3,300
	Width	580.0 ± 109.5	500 – 700
Oral sucker			
	Length	237.8 ± 20.3	223 – 260
	Width	196.8 ± 12.0	188 – 210
Ventral sucker			
	Length	175.4 ± 25.2	157 – 203
	Width	155.6 ± 35.1	130 – 194
Pharynx			
	Length	168.3 ± 32.7	109.7 – 182.9
	Width	109.7 ± 22.4	73.2 – 128.0
Anterior testis			
	Length	206.8 ± 14.8	196 – 223
	Width	155.2 ± 31.8	132 – 190
Posterior testis			
	Length	206.2 ± 56.4	165 – 268
	Width	194.2 ± 40.0	165 – 238
Ovary			
	Length	158.0 ± 19.2	144 – 179
	Width	103.2 ± 10.6	102 – 105
Egg			
	Length	27.2 ± 1.6	26 – 29
	Width	14.6 ± 2.1	13 – 17

Body small, oval and elongate, relatively stout, dorso-ventrally flattened, 1490–3300 (2214 ± 991.4) × 500–700 (580 ± 109.5) with a spinous tegument. Mid-body i.e., distance between the posterior margins of ventral sucker to the anterior margin of anterior testis, 645–1700 (1067 ± 577.8); oral sucker ventrosubterminal 223–260 (237.8 ± 20.3) × 188– 210 (196.8 ± 12.0). Ventral sucker located in anterior region of middle third of body, slightly smaller than oral sucker, 157–203 (175.4 ± 25.2) × 130–194 (155.6 ± 35.1) Prephaynx absent. Pharynx muscular and semi-circular, 109.7–182.9 (168.3 ± 32.7) × 73.2–128.0 (109.7 ± 22.4). Oesophagus absent. Intestine branches, run parallel to the body, extending anteriorly but not exceeding the pharynx and reaching close the posterior extremity of the body; all caeca exhibit sinuous diverticula. Testes large, oval, regular in form, tandem, with inter-testicular ovary, located in the posterior third of the body and extending near to posterior end, anterior testis 196–223 (206.8 ± 14.8) × 132–190 (155.2 ± 31.8); posterior testis 165–268 (206.2 ± 56.4) × 165–238 (194.2 ± 40.0).

Cirrus unarmed. Cirrus-pouch long and slender located anterior to anterior testis containing convoluted ejaculatory duct. Pars-prostatica short and surrounded by glandular cells. Seminal vesicle long, broad, saccular and unipartite. Smooth circular swelling surrounding the genital pore. Genital pore ventral, submedial, slightly dextral, anterior to anterior testis. Ovary lobed and regular in form, median located between testes, 144–179 (158.0 ± 19.2) × 102–105 (103.2 ± 10.6). Oviduct connects with seminal receptacle prior to ootype, surrounded by Mehlis’ gland. Uterus extending anteriorly but not exceeding the pharynx, coiled, between caeca. Metraterm present, apparently unarmed, opens into genital atrium. Eggs oval, 26–29 (27.2 ± 1.6) × 13–17 (14.6 ± 2.1), and round in cross-section.

Vitellarium follicular; follicles arranged in dendritic, moniliform system extending throughout most of region between levels of *c.* 50% of ventral sucker and not exceeding anterior testis, occupying the middle third of the body. Lateral vitelline collecting ducts extend throughout length of vitellarium, uniting to form a fusiform vitelline reservoir ventrally to ovary. Excretory pore terminal; excretory vesicle short that diverges at the end of caeca.

## Discussion

A parasitic survey allowed us to report for the first time, in the Caspian Sea area, a digenean identified as *Brachylaima* sp. isolated from a stray dog. Specimens were assigned to the genus *Brachylaima* based on the elongated shape of the body, the genital pore and cirrus sac anterior to anterior testis, vitellarium in middle third of the body, oesophagus absent and gonads located in tandem near the posterior extremity (12). Among the Brachylaimidae, the genus *Brachylaima* is diverse containing several species with very similar morphology (10). However, despite a large number of species reported in the genus, only a few have been extensively described and a complete set of morphological measurements are available i.e., *B. ruminae, B. cribbi, B. mascomai, B. llobregatensis* and *B. aspersae*, (13, 8-11) (Table 2). The specimens found in the stray dog in Iran have a smaller average length than specimens described from the five mentioned species (11). In addition, specimens here described differ from other species, except for *B. cribbi* and *B. aspersae*, in the presence of a distinct pars prostatica, from *B. aspersae* in the regular oval form of testes, and from *B. cribbi* in that vitelline follicle exceeds the posterior margin of ventral sucker (9, 11). However, describing species of *Brachylaima* using solely morphoanatomical and morphometric characteristics of the adult stages would be incomplete, as those features do not provide enough information on the species’ diversity, and information on the life cycle and developmental stages would be needed (9, 11). Thus, to be conservative, we rather prefer to leave this report as *Brachylaima* sp.

**Table 2:** Mean ± SD (range) of morphological measurements of *Brachylaima* sp. compared to other four species of *Brachylaima*. Measurements are given as length × width in micrometers unless otherwise stated.

	***Brachylaima sp. n = 5***	***B. mascomai n = 56***	***B. cribbi n = 30***	***B. llobregatensis n = 10***	***B. aspersae n. sp. n = 36***
	ex. *Canis familiaris*	ex. *Ratus norvegicus*	ex. *Mus musculus*	ex. *Mus musculus*	ex. *Mus musculus*
	This study	(8)	(9)	(10)	(11)
**Worm body**	2.21 ± 0.99 mm (1.5–3.3) × 0.58 ± 0.1 mm (0.5–0.7)	3.49 ± 0.52 mm (2.90–4.97) × 0.44 ± 0.04 mm (0.38–0.53)	5.00 mm (3.8–6.0) × 0.68 mm (0.52–0.79)^*^	3.39 ± 0.18 mm (2.08–2.73) × 0.62 ± 36.3 mm (0.56–0.68)	2.09 ± 0.29 mm (1.42–2.66) × 0.7 ± 0.07 mm (0.51–0.83)
**Oral Sucker**	237.8 ± 20.3 (223–260) × 196.8 ± 12.0 (188–210)	237.8 ± 21.5 (197–289) × 218.3 ± 23.5 (180–281)	259 (230–290) × 279 (250–380)^*^	235.9 ± 15.9 (2113–265) × 204–8 ± 16.1 (188.8–233.2)	248.2 ± 17 (200–275.5) × 222.2 ± 20.1 (167.5–255)
**Ventral Sucker**	175.4 ± 25.2 (157–203) × 155.6 ± 35.1 (130–194)	218.2 ± 21.6 (181–265) × 207.7 ± 20.3 (168–253)	277 (240–320) × 267 (230–300)^*^	223.1 ± 19.3 (181–241) × 216.1 ± 22.9 (168.8–237.4)	258.1 ± 17.3 (215–297.5) × 242.6±18.3 (187.5–270)
**Pharynx**	168.3±32.7 (109.7–182.9) × 109.7±22.4 (73.2–128.0)	116.9 ± 15.7 (84–164) × 148.7 ± 17.3 (105–180)	157 (140–180) × 169 (150–220)^*^	117.4 ± 3.4 (112.5–120.6) × 162.3 ± 6.4 (152.7–172.8)	172.2 ± 13.2 (137.5–195) × 133.8 ± 10.2 (115–157.5)
**Anterior testis**	206.8 ± 14.8 (196–223) × 155.2 ± 31.8 (132–190)	297.4 ± 26.4 (236–378) × 251.7 ± 37.0 (192–321)	419 (280–495) × 353 (240–450)^*^	279.4 ± 26.2 (241.2–321.6) × 241.7 ± 27.8 (180.9–265.3)	261.6 ± 38.2 (57.5–350) × 189 ± 38.7 (125–262.5)
**Posterior testis**	206.2 ± 56.4 (165–268) × 194.2 ± 40.0 (165–238)	319.1 ± 52.1 (239–422) × 247.7 ± 37.3 (188–336)	417 (250–530) × 323 (200–420)^*^	304.5 ± 32.7 (253.2–357.7) × 269.8 ± 29.8 (221.1–305.5)	273.6 ± 40 (195–355) × 216.5 ± 37.6 (145–285)
**Ovary**	158.0 ± 19.2 (144–179) × 103.2 ± 10.6 (102–105)	180.7 ± 31.1 (97–241) × 152.1 ± 21.8 (112–221)	217 (150–260 × 261 (170–320)^*^	191.9 ± 12.1 (172.8–209.1) × 125.1 ± 22.3 (88.4–160.8)	173.9 ± 28.6 (120–267.5) × 132.3 ± 22.1 (87.5–187.5)
**Egg**	27.2 ± 1.6 (26–29) × 14.6 ± 2.1 (13–17)	25.4 ± 0.8 (23–27.5) × 12.7 ± 0.2 (12.5–16)	29.1 (26.32) × 16.6 (16–17.5)^*^	30.9 ± 1.0 (29.3–32.5) × 18.2 ± 0.5 (17.6–18.8)	33.3 ± 1.1 (31–35) × 20.2 ± 2 (18–25)

* SD not provided by the authors.

Species of *Brachylaima* mostly occur in birds and mammals, and rodents are considered their main definitive hosts (6, 14). Only a few species have been reported in carnivores (15-22), and in only two cases, unidentified *Brachylaima* has been found in dogs from the North of Spain (23) and from the Khorasan Province in Northeast Iran (5). Most of the species of *Brachylaima* have been described from the Mediterranean region, and reports from other geographical places are considered introductions, probably imported from Europe (9, 14). Species of *Brachylaima* use land snails as first and second intermediate hosts (24). So, the economic trade of helicid snails would provide opportunities for species dissemination in a geographical context (14, 24). In Mazandaran Province, helicid snails coming from Europe are commonly found (25). Therefore, we raise the hypothesis that stray dogs might be infected with parasites through the consumption of helicid snails when searching for food in the street, and accordingly to the physiological characteristics of these mammals, worms would be able to fully complete their development.

For more than the last decade, only two species of digeneans have been reported in stray dogs in Iran through sporadic helminthic surveys (26). Moreover, a single record of *Brachylaima* sp. has been reported from dogs in Northeast Iran (5). Therefore, we contribute to the inventory of helminths in Iran and provide morphometric information for a *Brachylaima* species. We encourage researchers to place close attention on digeneans in future helminthic surveys in Iran, as for instance, gastrointestinal pathologies in humans have been found to be related to infections of mature *Brachylaima* species (9, 27).

## Conclusion

This is the second report of a species of *Brachylaima* in Iran and the third digenean species from stray dogs in the area. We want to raise the attention of researchers to helminthic surveys in potential zoonotic reservoirs like stray dogs.
